# Chaperone Copolymer Assisted G-Quadruplex-Based Signal Amplification Assay for Highly Sensitive Detection of VEGF

**DOI:** 10.3390/bios12050262

**Published:** 2022-04-20

**Authors:** Jialun Han, Chenxin Fang, Ping Ouyang, Yang Qing, Yuxing Yang, Haiyu Li, Zhencui Wang, Jie Du

**Affiliations:** College of Materials Science and Engineering, Hainan University, Haikou 570228, China; 18085204210013@hainanu.edu.cn (J.H.); 21220856000011@hainanu.edu.cn (C.F.); otping@hainanu.edu.cn (P.O.); 20080500210023@hainanu.edu.cn (Y.Q.); 20085600210065@hainanu.edu.cn (Y.Y.); 20080500110012@hainanu.edu.cn (H.L.); 20080500110014@hainanu.edu.cn (Z.W.)

**Keywords:** signal amplification, chaperone copolymer, G-quadruplex, VEGF detection

## Abstract

Vascular endothelial growth factor (VEGF) is a critical biomarker in the angiogenesis of several cancers. Nowadays, novel approaches to rapid, sensitive, and reliable VEGF detection are urgently required for early cancer diagnosis. Cationic comb-type copolymer, poly(L-lysine)-*graft*-dextran (PLL-*g*-Dex) accelerates DNA hybridization and chain exchange reaction while stabilizing the DNA assembly structure. In this work, we examined the chaperone activity of PLL-*g*-Dex to assist G-quadruplex-based fluorescent DNA biosensors for sensitive detection of VEGF. This convenient and effective strategy is based on chitosan hydrogel, c-myc, Thioflavin T (ThT), VEGF aptamer, and its partially complementary strand. The results show that chaperone copolymer PLL-*g*-Dex significantly promotes the accumulation of G-quadruplex and assembles into G-wires, allowing an effective signal amplification. Using this method, the detection limit of VEGF was as low as 23 pM, better than many previous works on aptamer-based VEGF detection. This chaperone copolymer-assisted signal amplification strategy has potential applications in the highly sensitive detection of target proteins, even including viruses.

## 1. Introduction

Vascular endothelial growth factor (VEGF) is a substance delivered by cells, simulating the formation of new blood vessels in tissues [1,2]. It is often overexpressed in the process of tumor growth and affects cancer metastasis due to its abnormally rapid growth and division. In this way, VEGF has been proved to be a critical biomarker in the angiogenesis of several cancers [3,4]. Recently, different kinds of detection strategies have been reported, such as luminescence assays [5], fluorescence detection [6,7,8], colorimetric test [9], and surface plasmon resonance [10]. However, these methods are time-consuming, expensive, and lack sensitivity. Therefore, rapid, accessible, and highly accurate VEGF detection biosensors are urgently required for early cancer diagnosis [11].

To date, owing to the high specificity and affinity, aptamer-based detection techniques have been widely developed [12,13,14,15,16]. However, in almost all of the aptamer-based technologies, without a signal amplification strategy, a one-to-one relationship between an aptamer and its correspondence target leads to low sensitivity and a high error rate. Thus, various signal amplification strategies have been widely explored, such as hybrid chain reaction (HCR), enzyme-assisted strand displacement reaction (EASD), and rolling circle amplification (RCA) [17,18,19,20,21,22]. Although these amplification strategies improve the sensitivity and lower the detection limit, they also have some potential obstacles. The HCR technique is limited by the hybridization kinetics of complementary strands and toehold exchange. It needs a high concentration of DNA or long sticky ends to retain the fast dynamic reaction [23,24,25]. In addition, due to the restriction of enzyme sensitivity, such as temperature and pH, the reaction conditions of ESDA and RCA must be strictly controlled [26,27]. Consequently, there is a great need to further develop a simple, rapid, and sensitive signal amplification strategy.

G-quadruplex is a secondary structure formed from a guanine-rich sequence, stabilized by the Hoogsteen-type pairs between guanine bases [28]. Because of both target recognition and signal transduction, and due to the ability to bind specific metal ions, dyes, enzymes, and proteins, G-quadruplex-based biosensors have attracted increasing interest in recent years [29,30,31,32,33]. Further, in the presence of K^+^ (promoting the formation of initial parallel stranded G-quadruplex) and Mg^2+^ (forming Mg-O coordination bond with DNA phosphate oxygen atom, neutralizing the negative charge of DNA and promoting the accumulation of G-quadruplex), G-quadruplex can assemble into G-quadruplex nanowires (G-wires) through π-π stacking interaction. Thus, the self-assembled G-wires can be applied as a signal amplifier to design optical and electrochemical biosensors [34,35].

Previously, assisted by the signal amplification strategy, we constructed a series of DNA fluorescent and electrochemical biosensors for sensitive detection of platelet-derived growth factor (PDGF-BB) [36], insulin [37], microRNA [38,39], adenosine triphosphate (ATP) [40,41,42], and streptavidin [43]. Our group also developed an artificial molecular chaperone for nucleic acids. A cationic comb-type copolymer poly(L-lysine)-*graft*-dextran (PLL-*g*-Dex) accelerates DNA hybridization and chain exchange reaction while stabilizing the multilevel DNA structure [44].

In this work, we examined the chaperone activity of CCC to assist G-quadruplex-based fluorescent DNA biosensors for sensitive and recyclable detection and extraction of VEGF. The purpose of this study was to design a simple, rapid, cost-effective, and sensitive assay for biomolecule detection with a non-labeled and enzyme-free DNA probe. This convenient and effective strategy is based on nanogel, c-myc, Thioflavin T (ThT), VEGF aptamer, and its partially complementary strand. In addition, chaperone copolymer PLL-*g*-Dex significantly promotes the accumulation of G-quadruplex and assembles into G-wires, allowing an effective signal amplification mediator for highly sensitive detection of target molecules.

## 2. Materials and Methods

### 2.1. Reagents and Materials

#### 2.1.1. Materials

All DNA oligomers were purchased from Bioengineering Co., Ltd. (Shanghai, China), listed in Table 1. Chitosan was provided by Solabao Co., Ltd. (Beijing, China). Chloropropene and epichlorohydrin were purchased from Sinopharm Chemical Reagent Co., Ltd. (Shanghai, China). VEGF was purchased from Boasen Biotechnology Co., Ltd. (Beijing, China). Ultrapure water was purchased from Dongsheng Biotech Co., Ltd. (Guangzhou, China). Other chemical reagents were purchased from Sigma-Aldrich (Shanghai, China). Quantikine^®^ QuicKit™ ELISAs were purchased from R&D Systems (Shanghai, China).

#### 2.1.2. Instrumentation

Fluorescence measurements were performed with a Model RF-6000 fluorescence spectrophotometer (Shimadzu, Kyoto, Japan). The fluorescence dye used was FAM with excitation and emission wavelengths of 494 nm and 522 nm, respectively. The wavelength range for spectral scanning was 494–560 nm. Circular dichroism (CD) spectra were measured on a Chirascan VX CD spectropolarimeter (Applied Photophysics Ltd., Surrey, UK). Each spectrum was collected from 200–300 nm at a scan rate of 100 nm min^−^^1^ and a response time of 2 s.

### 2.2. Experimental Procedures

#### 2.2.1. Synthesis of PLL-*g*-Dex

Cationic comb-type copolymer PLL-*g*-Dex was prepared by a reductive amination reaction of PLL·HBr (*M*_n_ = 20,000) with dextran (*M*_n_ = 5900, Dextran T-10) as described previously [45]. The dextran content of the copolymer was 91 wt%, determined by ^1^H NMR (Figure S1).

#### 2.2.2. Synthesis of Chitosan Hydrogel Film

The mass ratio of KOH/LiOH/urea/H_2_O is 6.5:5:7:81.5 to dissolve chitosan. Chitosan was dispersed into the above solution and all mixtures were stirred at 30 °C for 12 h. Three milliliters of chloropropene was added into fifty grams of chitosan solution (3.5 wt%) and then stirred at 0 °C away from light for 12 h to obtain allyl chitosan (AC), as shown in Figure S2A. Epichlorohydrin was added to AC to crosslink it into hydrogels. The hydrogel was dried to obtain AC hydrogel film (ACF), as shown in Figure S2B.

#### 2.2.3. Sulfydryl-DNA Grafted into CHITOSAN Hydrogel Film

The chitosan film was immersed in 500 μL in different concentrations of (2 μM, 4 μM, 6 μM, 8 μM, 10 μM) sulfydryl-DNA1 with 0.05 wt% photoinitiator I2959. It was then irradiated with ultraviolet light (365 nm) for 5 min. The films were immersed in 500 μL ultrapure water for 2 h to remove unreacted molecules.

## 3. Results and Discussion

**Figure 1:**
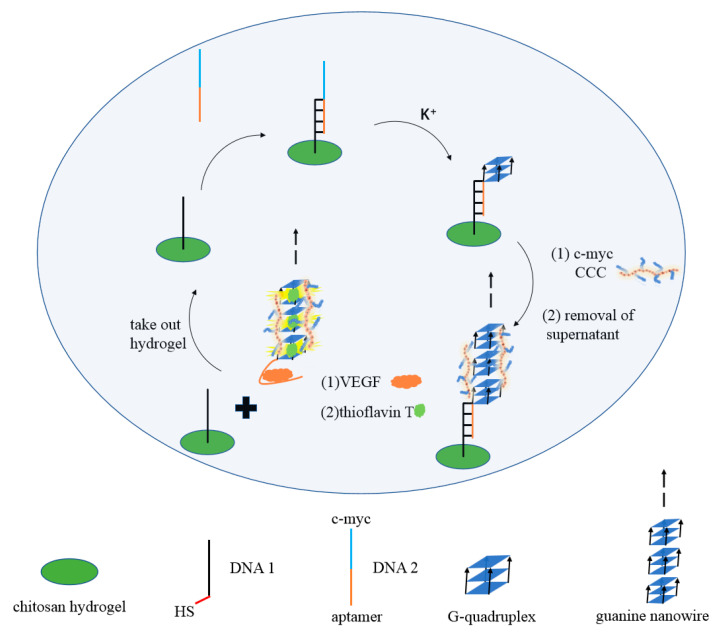
Schematic illustration of the fluorescence assay for the detection of VEGF.

Figure 1 shows the operating principle of the G-quadruplex-based fluorescent DNA biosensor for sensitive and recyclable detection of VEGF. Firstly, the sulfhydryl modified DNA1 was grafted onto the chitosan hydrogel film via click chemistry reaction. Then, DNA2 containing c-myc and VEGF aptamer was added to form the G-quadruplexes in the presence of K^+^. Next, chaperone copolymer PLL-*g*-Dex significantly promotes the accumulation of G-quadruplex and assembles into G-wires. Upon the addition of VEGF, the formation of the aptamer/VEGF complex was induced, and the G-wires were released from the chitosan hydrogel film. After centrifugation, the specific dye ThT was added to the upper clear solution, and G-wires combined with ThT to produce a strong fluorescence signal. In the absence of VEGF, G-wires could not be released from the hydrogel film, resulting in weak fluorescence. Using this strategy, VEGF can be detected quantitatively.

**Figure 2:**
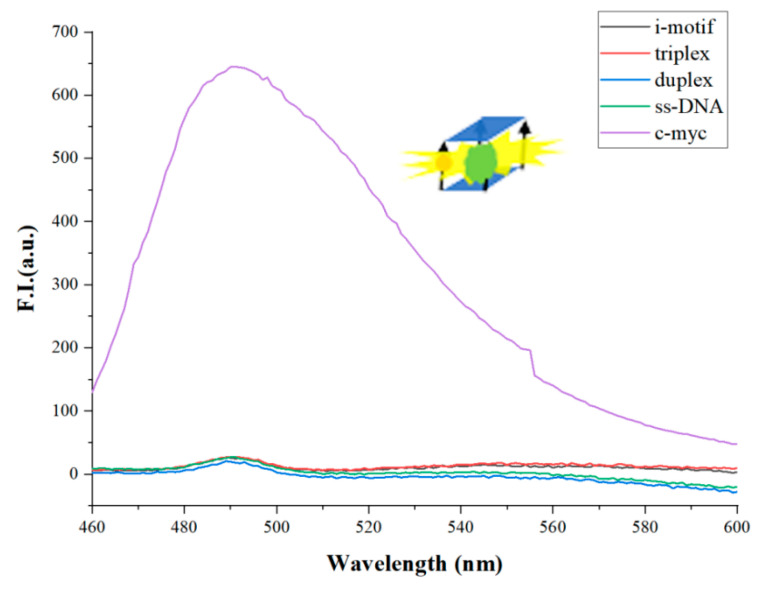
The fluorescence intensity of different DNA structures (i-motif, triplex, duplex, ss-DNA, c-myc). ([ThT] = 2 μM, [K^+^] = 50 mM. The concentration of each oligonucleotide is 3 μM).

At first, we compared G-quadruplex to other oligomers to test the specificity binding of ThT to G-quadruplex. Figure 2 shows the fluorescence spectra of some oligomers, such as i-motif, triple-stranded DNA, double-stranded DNA, single-stranded DNA, and G-quadruplex (c-myc). The concentration of each oligonucleotide is 3 μM. In the presence of K^+^ (50 mM), c-myc folded into the G-quadruplex structure, while the dye ThT (2 μM) specifically embedded into the G-quadruplex to emit fluorescence. We set the wavelength range from 460 nm to 600 nm. Λ = 490 nm is the emission wavelength of ThT. Except for c-myc, the fluorescence intensity of other oligomers is very weak. Under the same experimental conditions, the fluorescence intensity of c-myc is more than 40 times that of these oligomers. Therefore, we confirm that K^+^ can help c-myc fold into G-quadruplex, and ThT specifically enters G-quadruplex to emit strong fluorescence at λ = 490 nm. Therefore, this method can be used to construct a new method to distinguish DNA with other topological structures from parallel stranded G-quadruplexes.

**Figure 3:**
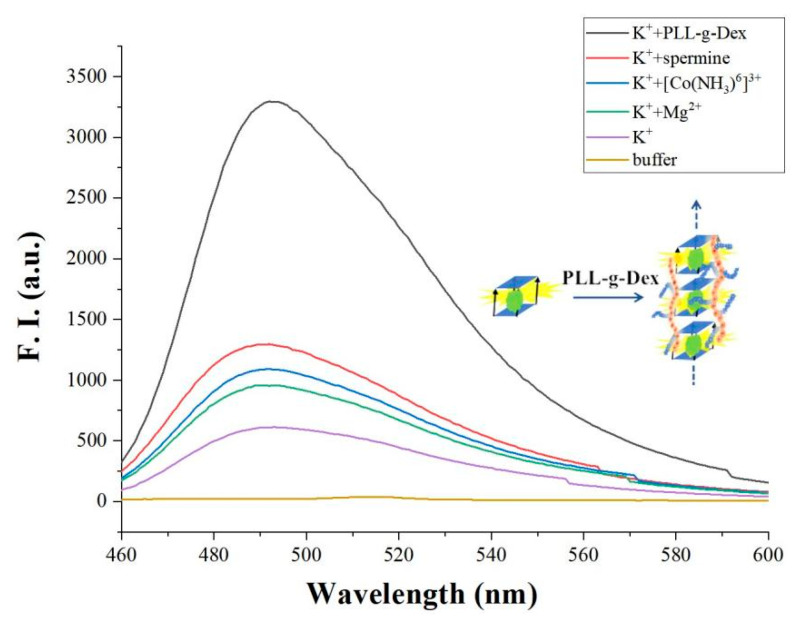
The fluorescence intensity of adding different cations ([c-myc] = 2 μM, [ThT] = 2 μM, [K^+^] = 50 mM, [Mg^2+^] = 1 mM, [Co(NH_3_)_6_]^3+^ = 1 mM, spermine = 1 mM, PLL-*g*-Dex = 0.1 μM).

As mentioned above, K^+^ promotes the formation of an initial parallel G-quadruplex. Further, Mg^2+^ forms Mg-O coordination bond with DNA phosphate oxygen atom, neutralizing the negative charge of DNA and promoting the accumulation of G-quadruplex. Thus, in the presence of Mg^2+^, G-quadruplex assembles into G-wires through π-π stacking interaction. As shown in Figure 3, we confirmed that, in the presence of Mg^2+^ (1 mM), the fluorescence value of G-quadruplex further increases. The reason is that the formation of G-wires enables more G-quadruplex to link with the biosensors matrix, thereby amplifying the signal. Note that other cations, such as [CO (NH_3_)_6_]^3+^ and arginine, can also promote the formation of G-wires from G-quadruplex. In Figure 3, compared with Mg^2+^, the same concentration (1 mM) of [CO (NH_3_)_6_]^3+^ or arginine can further increase the fluorescence intensity of G-quadruplex. It may be that [CO (NH_3_)_6_]^3+^ or arginine with multivalent positive charge makes G-wires more stable than magnesium ions with divalent charge does. Therefore, we concluded that proper cations can assist the formation of G-wires. From this point of view, we tested cationic comb-type copolymer PLL-*g*-Dex to accelerate the formation of G-wires. The interesting result shows that, with a much lower concentration of PLL-*g*-Dex (0.1 μM), the fluorescence intensity of G-quadruplex is significantly enhanced. The reason is similar to our previous work, that is, PLL-*g*-Dex reduces the adverse anti-ion condensation effect of entropy and reduces the energy barrier related to the breaking and recombination of nucleic acid base pairs. Although the interaction is weakened, the cationic copolymer can still inhibit the repulsion between DNA strands, which is enough to stabilize the multilevel DNA structure [43]. In addition to the shielding effect on repulsion, the Dex chain may also play a role in stabilizing the hydrogen bond between base pairs. DNA attracted to the PLL backbone through electrostatic interaction is forced to merge with Dex enrichment with a low dielectric constant. This low dielectric environment may enhance the hydrogen bond between base pairs and stabilize G-wires.

**Figure 4:**
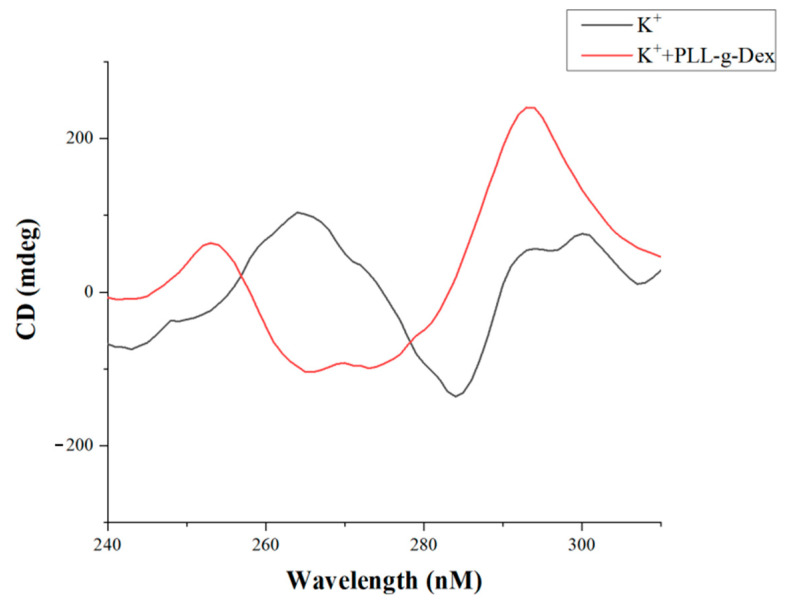
Experimental results of CD ([K^+^] = 50 mM, [PLL-*g*-Dex] = 0.1 μM, [c-myc] = 2.0 μM).

The formation of G-quadruplex in the sensing system was further confirmed by CD spectroscopy (Figure 4). Adding K^+^, the CD spectrum shows a positive peak at about 265 nm and a negative peak at about 240 nm, indicating the formation of a parallel G-quadruplex. Interestingly, when the cationic polymer PLL-*g*-Dex is added, the negative peak at 240 nm disappears, while the negative peak appears at 264 nm and the positive peak appears at 295 nm, which is the signal of antiparallel G-quadruplex. That means, PLL-*g*-Dex not only assembles G-quadruplex into G-wires but also changes the conformation of G-quadruplex.

**Figure 5:**
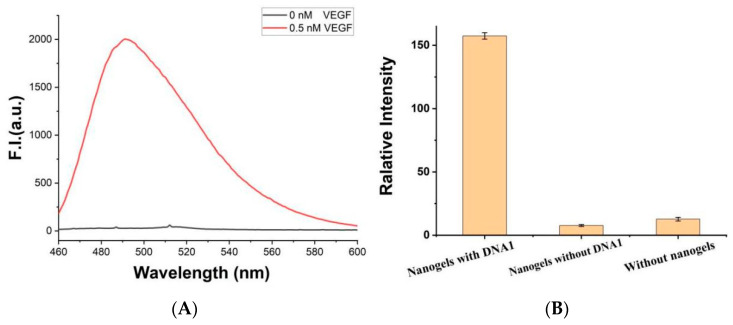
(**A**) Fluorescence spectrum of the platform ([ThT] = 2 μM, [gels] = 0.15 mg/mL, [K^+^] = 50 mM, [PLL-*g*-Dex] = 0.1 μM, [c-myc] = 2.0 μM) with (0.5 nM) or without VEGF. (**B**) Fluorescence signal of the VEGF (0.02 nM) detection system with or without DNA1, or without chitosan hydrogel. Error bars represent the standard deviation of the measurement (n = 3).

In this work, chitosan hydrogel modified by a double bond was firstly synthesized, as shown in Figure S2. Then, the thiol modified DNA1 was grafted onto the chitosan hydrogel film through click chemistry reaction. DNA2 containing c-myc and VEGF aptamer was then added into the system. The VEGF aptamer part hybrids with DNA1, and c-myc part forms the G-quadruplex under the action of K^+^. Further, in the presence of chaperone polymer PLL-*g*-Dex, G-quadruplex rapidly folds into extended G-wires. When the target protein VEGF is present, the complex of aptamer/VEGF is induced, and the G-wires are released from the gel. After centrifugation, specific dye ThT was added into the upper clear solution, and G-wires combined with ThT to produce a strong fluorescence signal. In the absence of VEGF, G-wires cannot be released from the gel, and fluorescence signals are relatively weak, as shown in Figure 5A. After centrifugation, the chitosan hydrogel was washed thoroughly with excess Milli-Q ultrapure water, and thus could be reused next time.

In order to test the detection performance, 1 nM of VEGF was introduced into the biosensor system containing 0.15 mg mL^−1^ of chitosan hydrogel. After incubation at room temperature for 20 min, K^+^ (50 mM), c-myc (2.0 μM) and PLL-*g*-Dex (0.1 μM) were added into the system. Interestingly, enhanced fluorescence intensity was observed in the presence of VEGF. However, without DNA1 or chitosan hydrogel, fluorescence intensities were very weak, as shown in Figure 5B, indicating that the increase in ThT fluorescence is not due to the direct interaction between ThT and gel or VEGF. The enhancement of fluorescence intensity is due to the specific binding of VEGF and aptamer chain, leading to the separation of G-wires from the gel into the solution. Therefore, the feasibility of this experimental principle is confirmed.

In order to confirm thiol modified DNA1 can be integrated into chitosan hydrogel, the chitosan film was immersed in FAM and thiol-labeled DNA1 (cDNA), and FAM-labeled DNA1, respectively. Then, it was irradiated with ultraviolet light for 5 min with 0.05 wt% I2959, via click chemical reaction for grafting. The gel was washed in buffer solution to remove unreacted DNA1, observed by fluorescence microscope. The blank control group was only chitosan hydrogel without DNA1, no fluorescence was detected (Figure S3a). cDNA labeled with both FAM and thiol was grafted onto the gel, showing strong fluorescence intensity (Figure S3b). Without the sulfydryl group FAM-labeled DNA1 alone can not react with the double bond in chitosan hydrogel, and thus no fluorescence will be detected (Figure S3c). The results showed that thiol-labeled DNA1 can be successfully integrated into the carrier by clicking chemical reactions.

**Figure 6:**
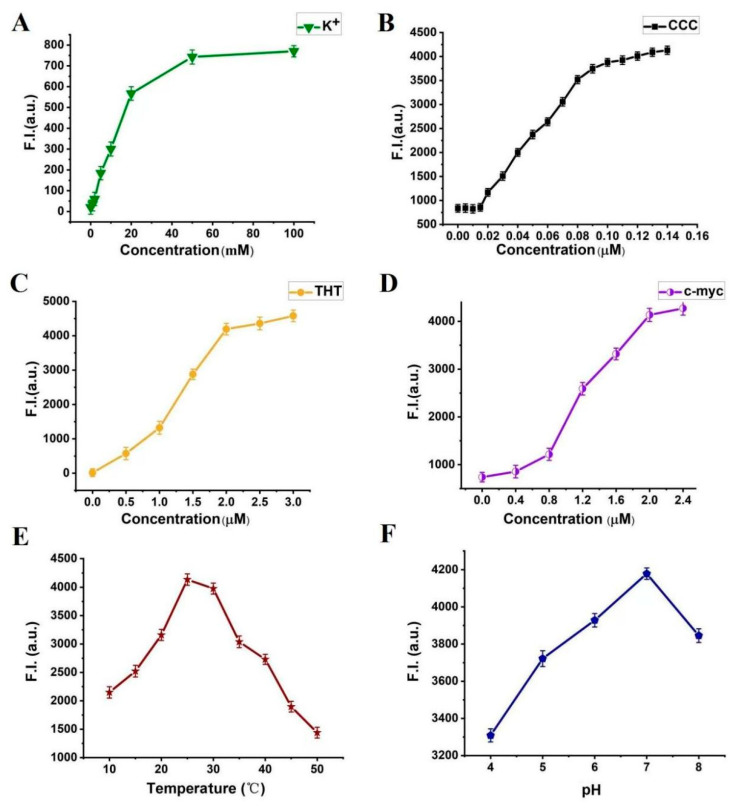
Fluorescence intensity of the system. Error bars represent the standard deviation of the measurement (n = 3). (**A**) different concentrations of K^+^ (0–100 mM); (**B**) different concentrations of PLL-*g*-Dex (0.005–0.14 µM); (**C**) different concentrations of ThT (0–3 µM); (**D**) different concentrations of c-myc (0–2.4 µM); (**E**) different temperatures (10–50 °C); (**F**) different pH (4, 5, 6, 7 and 8).

In order to make the biosensor system more sensitive, we optimized the experimental conditions by changing the variables listed as follows: concentration of K^+^: 0, 1, 2, 5, 10, 20, 50, and 100 mM; concentration of PLL-*g*-Dex: 0.005, 0.010, 0.015, 0.020, 0.030, 0.040, 0.050, 0.060, 0.070, 0.080, 0.090, 0.100, 0.110, 0.120, 0.130, and 0.140 μM; concentration of ThT: 0, 0.5, 1.0, 1.5, 2.0, 2.5, and 3.0 μM; concentrations of c-myc: 0, 0.4, 0.8, 1.2, 1.6, 2.0, and 2.4 μM; temperatures: 10, 15, 20, 25, 30, 35, 40, 45, and 50 °C; pH: 4, 5, 6, 7, and 8. Without target, the optimized condition is used to carry out the following experiment. The fluorescence intensity is used as the baseline of the target detection. The results showed that a highly sensitive VEGF detection platform was constructed under the following conditions: K^+^ (50 mM), PLL-*g*-Dex (0.1 μM), ThT (0.2 μM), c-myc (2.0 μM), temperature (25 °C), and pH 7.0, as shown in Figure 6.

**Figure 7:**
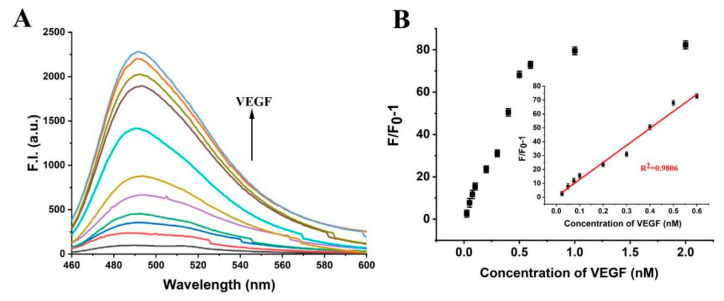
(**A**) Fluorescence spectra of the platform with 0.025, 0.05, 0.075, 0.1, 0.2, 0.3, 0.5, 1.0, 1.5, and 2.0 nM of PDGF-BB. (**B**) The relationship between fluorescence intensity at λ = 490 nm and VEGF concentrations. Inset: The standard curve of VEGF detection. Error bars represent the standard deviation of the measurement (n = 3).

We carried out the detection of VEGF concentration under the optimized conditions. Reasonably, the fluorescence signal of ThT increased with the increase in VEGF concentration (Figure 7A). The platform showed that the linear range detection of VEGF was 0.025–0.3 nM, and the maximum fluorescence intensity increased by about 17 times (Figure 7B). Using 3σmethods, the detection limit of VEGF was as low as 23 pM, better than many previous works on aptamer-based VEGF detection systems, listed in Table 2.

**Figure 8:**
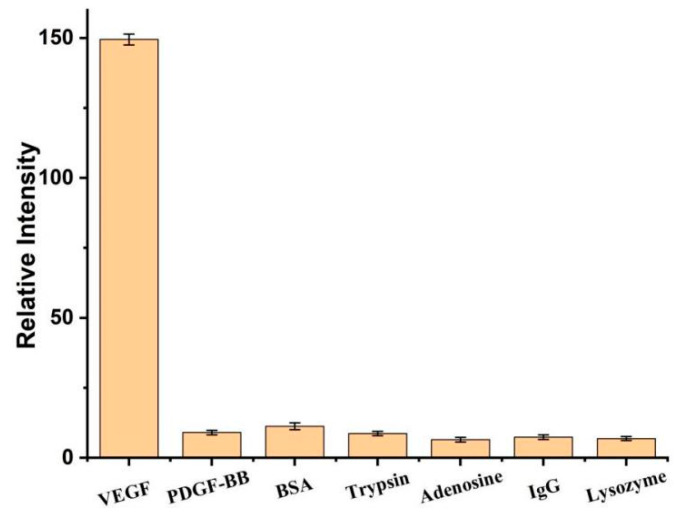
Relative fluorescence intensity of 0.1 nM VEGF or 10-fold excess amount of other proteins. Error bars represent the standard deviation of the measurement (n = 3).

Compared with other protein detection methods, such as platelet-derived growth factor (PDGF-BB), bovine serum albumin (BSA), trypsin, adenosine, immunoglobulin G (IgG), and lysozyme, the selectivity of VEGF detection assay was studied. The results showed that the fluorescence intensity of the VEGF platform is significantly higher than that of even a 10-fold excess amount of other proteins by more than 15 times (Figure 8). Compared with other proteins, the high selectivity of the VEGF platform is attributed to the specific binding between VEGF and its aptamer.

To determine whether the system is suitable for biological matrix, we tested 1% (*v*/*v*) human serum samples with different VEGF concentrations, and the results are listed in Table 3 (outside parentheses). Using the proposed method, we obtained a recovery of about 99.71% in human serum samples. The recovery (between 98.46% and 100.69%) and relative standard deviation (RSD) (between 0.1% and 3.24%) are feasible. The results showed that the detection platform has potential advantages in the analysis and detection of complex biological samples. At present, antibody-based detection methods such as enzyme-linked immunosorbent assay (ELISA) are the only commercially available VEGF detection kits. The data in parentheses were measured by ELISA, as listed in Table 3. Compared with the proposed method in this work, ELISA-based detection has many disadvantages, such as expensive antibodies, long incubation time, and complicated schemes. More importantly, compared with the chaperone copolymer-assisted signal amplification strategy, the sensitivity of ELISA is quite limited. Therefore, according to the data measured by chaperone copolymer-assisted G-quadruplex-based biosensor and compared with the current commercial VEGF detection kit, the proposed method is more rapid, sensitive, and reliable. In addition, different from the antibody-based detection protocol, the chaperone copolymer-assisted signal amplification strategy can become a promising tool in early cancer diagnosis.

**Figure 9:**
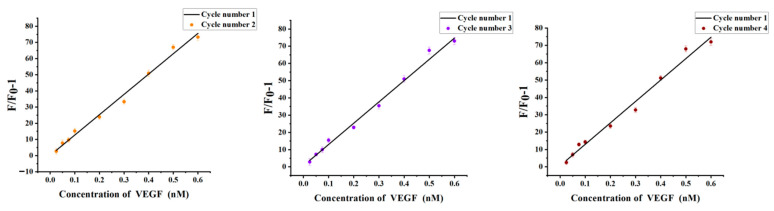
Comparison of the standard curves of the four cycles. Error bars represent the standard deviation of the measurement (n = 3).

The chitosan hydrogel was collected by centrifugation and redispersed in the new solution. After washing with excess Milli-Q ultrapure water, the hydrogel was collected and recycled. 0.025–0.6 nM of VEGF was added to verify the sensitivity of the recovered hydrogel. The results showed that the fluorescence intensity in 490 nm combined with the above standard curve equation, indicating that the hydrogel has almost recovered to its original structure. In addition, there was no significant difference in the standard curve of the four cycles, as shown in Figure 9. So, chitosan hydrogel can be used as a carrier many times.

## 4. Conclusions

We developed a novel method for rapid, sensitive, and reliable VEGF detection. This method is based on chitosan hydrogel, c-myc, Thioflavin T (ThT), VEGF aptamer, and its partially complementary strand. Chaperone copolymer PLL-*g*-Dex significantly promotes G-quadruplex assembled into G-wires. VEGF binds with aptamer, and the G-wires are released from the chitosan hydrogel film. After centrifugation, G-wires combined with ThT to produce a strong fluorescence signal. Using this strategy, VEGF can be detected sensitively. The platform shows that the linear range detection of VEGF is 0.025–0.3 nM and the detection limit of VEGF is as low as 23 pM, better than many previous works on aptamer-based VEGF detection. This chaperone copolymer-assisted signal amplification strategy has potential applications in early cancer diagnosis.

## Figures and Tables

**Table 1:** Sequences of the oligonucleotides used in this study.

Name	Sequence (from 5′ to 3′)	Length (nt)
DNA1	SH-CACTGAGTCCCTGCACTCTTGTCTGGAAGACGGG	34
DNA2	AGGGTGGGGAGGGTGGGGCCCGTCTTCCAGACAAGAGTGCAGGG	44
c-myc	AGGGTGGGGAGGGTGGGG	18
cDNA	SH-CACTGAGTCCCTGCACTCTTGTCTGGAAGACGGG-FAM	34

**Table 2:** Comparison of the proposed assay with other published methods for VEGF detection.

Detection Method	Strategy	Linear Range	Detection Limit	Refs.
Luminescence	Aptamer controlled catalysis of a Porphyrin Probe	0–25 nM	50 pM	[5]
Fluorescence	Fluorescence polarization based on recognition reaction	0.32–5.0 nM	0.32 nM	[6]
Fluorescence	Microchip electrophoresis	5.00–150.0 nM	2.48 nM	[7]
Fluorescence	PNA bound/free separation system	5–50 nM	25 nM	[8]
Colorimetric	Strand displacement amplification	24.00 pM to 11.25 nM	1.70 pM	[9]
Surface Plasmon Resonance	Plastic optical fiber (POF)-SPR	-	3 nM	[10]
Fluorescence	Chaperone copolymer-assisted signal amplification	0.025–0.3 nM	23 pM	This work

**Table 3:** Detection of VEGF in 1% (*v*/*v*) human serum with the proposed method (outside parentheses) and with Quantikine^®^ ELISA (in parentheses).

Sample No.	Added (pM)	Found (pM)	Recovery (%)	RSD (%, n = 4)	Assay Time
1	50.00	49.23 (48.51)	98.46 (97.02)	3.24 (4.35)	5 min (4.5 h)
2	75.00	74.08 (72.64)	98.77 (96.85)	0.78 (4.02)	
3	100.00	100.50 (96.32)	100.50 (96.32)	0.10 (3.38)	
4	200.00	200.33 (196.48)	100.16 (98.24)	0.59 (4.26)	
5	500.00	502.96 (484.25)	100.59 (96.85)	1.07 (5.67)	

## Data Availability

Not applicable.

## Supplementary Materials

The following supporting information can be downloaded at: https://www.mdpi.com/article/10.3390/bios12050262/s1, Figure S1: (A) Structural formula of poly(L-lysine)-*graft*-dextran (PLL-*g*-Dex) copolymer. (B) ^1^H-NMR spectra of PLL, Dex and PLL-*g*-Dex in D_2_O. The dextran content of the copolymer was calculated from ^1^H-NMR signals assigned to PLL(ε-CH_2_) and dextran(C_1_-H, a).; Figure S2: (A) Allylation of chitosan in KOH/LiOH/urea solution. (B) Schematic representation of ECH cross-link AC to prepare hydrogel film; Figure S3: Fluorescent images the gels formed (a) without DNA1, (b) with FAM/SH-labeled DNA1, (c) with FAM-labeled DNA.

## References

[1] Li J., Sun K., Chen Z., Shi J., Zhou D., Xie G. (2017). A flfluorescence biosensor for VEGF detection based on DNA assembly structure switching and isothermal amplifification. Biosens. Bioelectron..

[2] Da H., Liu H., Zheng Y., Yuan R., Chai Y. (2018). A highly sensitive VEGF165 photoelectrochemical biosensor fabricated by assembly of aptamer bridged DNA networks. Biosens. Bioelectron..

[3] Dehghani S., Nosrati R., Yousefi M., Nezami A., Soltani F., Taghdisi S.M., Ramezani M. (2018). Aptamer-based biosensors and nanosensors for the detection of vascular endothelial growth factor (VEGF): A review. Biosens. Bioelectron..

[4] Zhao Z., Al-Ameen M.A., Duan K., Ghosh G., Lo J.F. (2015). On-chip porous microgel generation for microfluidic enhanced VEGF detection. Biosens. Bioelectron..

[5] Li W., Zhang Q., Zhou H., Chen J., Li Y., Zhang C., Yu C. (2015). Chemiluminescence detection of a protein through the aptamer-controlled catalysis of a porphyrin probe. Anal. Chem..

[6] Wang S.E., Huang Y., Hu K., Tian J., Zhao S. (2014). A highly sensitive and selective aptasensor based on fluorescence polarization for the rapid determination of oncoprotein vascular endothelial growth factor (VEGF). Anal. Methods.

[7] Lin X., Chen Q., Liu W., Yi L., Li H., Wang Z., Lin J.M. (2015). Assay of multiplex proteins from cell metabolism based on tunable aptamer and microchip electrophoresis. Biosens. Bioelectron..

[8] Mita C., Abe K., Fukaya T., Ikebukuro K. (2014). Vascular endothelial growth factor (VEGF) detection using an aptamer and PNA-based bound/free separation system. Materials.

[9] Zhang H., Peng L., Li M., Ma J., Qi S., Chen H., Zhou L., Chen X. (2017). A label-free colorimetric biosensor for sensitive detection of vascular endothelial growth factor-165. Analyst.

[10] Cennamo N., Pesavento M., Lunelli L., Vanzetti L., Pederzolli C., Zeni L., Pasquardini L. (2015). An easy way to realize SPR aptasensor: A multimode plastic optical fiber platform for cancer biomarkers detection. Talanta.

[11] Moradi R., Khalili N.P., Septiani N.L.W., Liu C.H., Doustkhah E., Yamauchi Y., Rotkin S.V. (2022). Nanoarchitectonics for Abused-Drug Biosensors. Small.

[12] Kim Y.S., Raston N.H.A., Gu M.B. (2016). Aptamer-based nanobiosensors. Biosens. Bioelectron..

[13] Zhou W., Huang P.J.J., Ding J., Liu J. (2014). Aptamer-based biosensors for biomedical diagnostics. Analyst.

[14] Park K.S. (2018). Nucleic acid aptamer-based methods for diagnosis of infections. Biosens. Bioelectron..

[15] Li F., Yu Z., Han X., Lai R.Y. (2019). Electrochemical aptamer-based sensors for food and water analysis: A review. Anal. Chim. Acta.

[16] Mehlhorn A., Rahimi P., Joseph Y. (2018). Aptamer-based biosensors for antibiotic detection: A review. Biosensors.

[17] Mittal S., Kaur H., Gautam N., Mantha A.K. (2017). Biosensors for breast cancer diagnosis: A review of bioreceptors, biotransducers and signal amplification strategies. Biosens. Bioelectron..

[18] Lei J., Ju H. (2012). Signal amplification using functional nanomaterials for biosensing. Chem. Soc. Rev..

[19] Liu L., Yang D., Liu G. (2019). Signal amplification strategies for paper-based analytical devices. Biosens. Bioelectron..

[20] Li F., Zhou Y., Yin H., Ai S. (2020). Recent advances on signal amplification strategies in photoelectrochemical sensing of microRNAs. Biosens. Bioelectron..

[21] Xu M., Tang D. (2021). Recent advances in DNA walker machines and their applications coupled with signal amplification strategies: A critical review. Anal. Chim. Acta.

[22] Abolhasan R., Mehdizadeh A., Rashidi M.R., Aghebati-Maleki L., Yousefi M. (2019). Application of hairpin DNA-based biosensors with various signal amplification strategies in clinical diagnosis. Biosens. Bioelectron..

[23] Dong J., Zeng Z., Sun R., Zhang X., Cheng Z., Chen C., Zhu Q. (2021). Specific and sensitive detection of CircRNA based on netlike hybridization chain reaction. Biosens. Bioelectron..

[24] Zhang K., Lv S., Zhou Q., Tang D. (2020). CoOOH nanosheets-coated g-C3N4/CuInS2 nanohybrids for photoelectrochemical biosensor of carcinoembryonic antigen coupling hybridization chain reaction with etching reaction. Sens. Actuators B Chem..

[25] Oishi M., Juji S. (2021). Acceleration of DNA Hybridization Chain Reactions on 3D Nanointerfaces of Magnetic Particles and Their Direct Application in the Enzyme-Free Amplified Detection of microRNA. ACS Appl. Mater. Interfaces.

[26] Zhang K., Yang L., Lu F., Wu X., Zhu J.J. (2018). A Universal Upconversion Sensing Platform for the Sensitive Detection of Tumour-Related ncRNA through an Exo III-Assisted Cycling Amplification Strategy. Small.

[27] Yang L., Fung C.W., Cho E.J., Ellington A.D. (2007). Real-time rolling circle amplification for protein detection. Anal. Chem..

[28] Yang H., Zhou Y., Liu J. (2020). G-quadruplex DNA for construction of biosensors. TrAC Trends Anal. Chem..

[29] Xi H., Juhas M., Zhang Y. (2020). G-quadruplex based biosensor: A potential tool for SARS-CoV-2 detection. Biosens. Bioelectron..

[30] Nishio M., Tsukakoshi K., Ikebukuro K. (2021). G-quadruplex: Flexible conformational changes by cations, pH, crowding and its applications to biosensing. Biosens. Bioelectron..

[31] Xu J., Jiang R., He H., Ma C., Tang Z. (2021). Recent advances on G-quadruplex for biosensing, bioimaging and cancer therapy. TrAC Trends Anal. Chem..

[32] Ahmadi Y., Soldo R., Rathammer K., Eibler L., Barišić I. (2021). Analyzing criteria affecting the functionality of G-quadruplex-based DNA aptazymes as colorimetric biosensors and development of quinine-binding aptazymes. Anal. Chem..

[33] Guo J., Feng C., Liu Z., Ye B., Li G., Zou L. (2021). A label-free electrochemical biosensor based on novel DNA nanotweezer coupled with G-quadruplex for sensitive DNA detection. Sens. Actuators B Chem..

[34] Xu Y., Lu Z., Fu X., Yu F., Chen H., Nie Y. (2020). Guanine-wire based walking machine for highly sensitive and selective detection of circulating microRNA. Sens. Actuators B Chem..

[35] Bi Q., Qiu F., Yuan R., Xiang Y. (2021). In situ formation of G-quadruplex/hemin nanowires for sensitive and label-free electrochemical sensing of acid phosphatase. Sens. Actuators B Chem..

[36] Zhang Z., Han J., Li Y., Du J. (2018). A sensitive and recyclable fluorescence aptasensor for detection and extraction of platelet-derived growth factor BB. Sens. Actuators B Chem..

[37] Liu C., Han J., Zhang J., Du J. (2019). Novel detection platform for insulin based on dual-cycle signal amplification by Exonuclease III. Talanta.

[38] Liu C., Han J., Zhou L., Zhang J., Du J. (2020). DNAzyme-based target-triggered rolling-circle amplification for high sensitivity detection of microRNAs. Sensors.

[39] Fang C., Ouyang P., Yang Y., Qing Y., Han J., Shang W., Chen Y., Du J. (2021). MiRNA Detection Using a Rolling Circle Amplification and RNA-Cutting Allosteric Deoxyribozyme Dual Signal Amplification Strategy. Biosensors.

[40] Zhang J., Yang C., Niu C., Liu C., Cai X., Du J., Chen Y. (2018). A label-free fluorescent and logic gate aptasensor for sensitive ATP Detection. Sensors.

[41] Zhang J., Han J., Feng S., Niu C., Liu C., Du J., Chen Y. (2018). A Label-Free Fluorescent DNA Machine for Sensitive Cyclic Amplification Detection of ATP. Materials.

[42] Zhang J., Zhang S., Niu C., Liu C., Du J., Chen Y. (2018). A label-free fluorescent DNA calculator based on gold nanoparticles for sensitive detection of ATP. Molecules.

[43] Ouyang P., Fang C., Han J., Zhang J., Yang Y., Qing Y., Chen Y., Shang W., Du J. (2021). A DNA Electrochemical Sensor via Terminal Protection of Small-Molecule-Linked DNA for Highly Sensitive Protein Detection. Biosensors.

[44] Du J., Wu L., Shimada N., Kano A., Maruyama A. (2013). Polyelectrolyte-assisted transconformation of a stem-loop DNA. Chem. Commun..

[45] Zhang Z., Wu Y., Yu F., Niu C., Du Z., Chen Y., Du J. (2017). Rapid and annealing-free self-assembly of DNA building blocks for 3D hydrogel chaperoned by cationic comb-type copolymers. J. Biomater. Sci. Polym. Ed..

