# Supplementary material for: Chaperone Copolymer Assisted G-Quadruplex-Based Signal Amplification Assay for Highly Sensitive Detection of VEGF
Source: Biosensors (Basel). 2022 Apr 20;12(5):262. doi: 10.3390/bios12050262 (PMC9138322; doi:10.3390/bios12050262)
Supplement: Supplementary file 1 [file biosensors-12-00262-s001.zip › biosensors-1685613-supplementary.pdf]

biosensors

MDPI

*Supporting Information*

# Chaperone Copolymer Assisted G-Quadruplex-Based Signal Amplification Assay for Highly Sensitive Detection of VEGF

**Jialun Han, Chenxin Fang, Ping Ouyang, Yang Qing, Yuxing Yang, Haiyu Li, Zhencui Wang, and Jie Du ***

College of Materials Science and Engineering, Hainan University, Haikou 570228, China; 18085204210013@hainanu.edu.cn (J.H.); 21220856000011@hainanu.edu.cn (C.F.); otping@hainanu.edu.cn (P.O.); 20080500210023@hainanu.edu.cn (Y.Q.); 20085600210065@hainanu.edu.cn (Y.Y.); 20080500110012@hainanu.edu.cn (H.L.); 20080500110014@hainanu.edu.cn (Z.W.)

* Correspondence: dujie@hainanu.edu.cn

**Citation:** Han, J.; Fang, C.; Ouyang, P.; Qing, Y.; Yang, Y.; Li, H.; Wang, Z.; Du, J. Chaperone Copolymer Assisted G-Quadruplex-Based Signal Amplification Assay for Highly Sensitive Detection of VEGF. *Biosensors* **2022**, *12*, 262. https://doi.org/10.3390/bios12050262

Received: 31 March 2022

Accepted: 19 April 2022

Published: 20 April 2022

**Publisher's Note:** MDPI stays neutral with regard to jurisdictional claims in published maps and institutional affiliations.

**Copyright:** © 2022 by the authors. Licensee MDPI, Basel, Switzerland. This article is an open access article distributed under the terms and conditions of the Creative Commons Attribution (CC BY) license (https://creativecommons.org/licenses/by/4.0/).

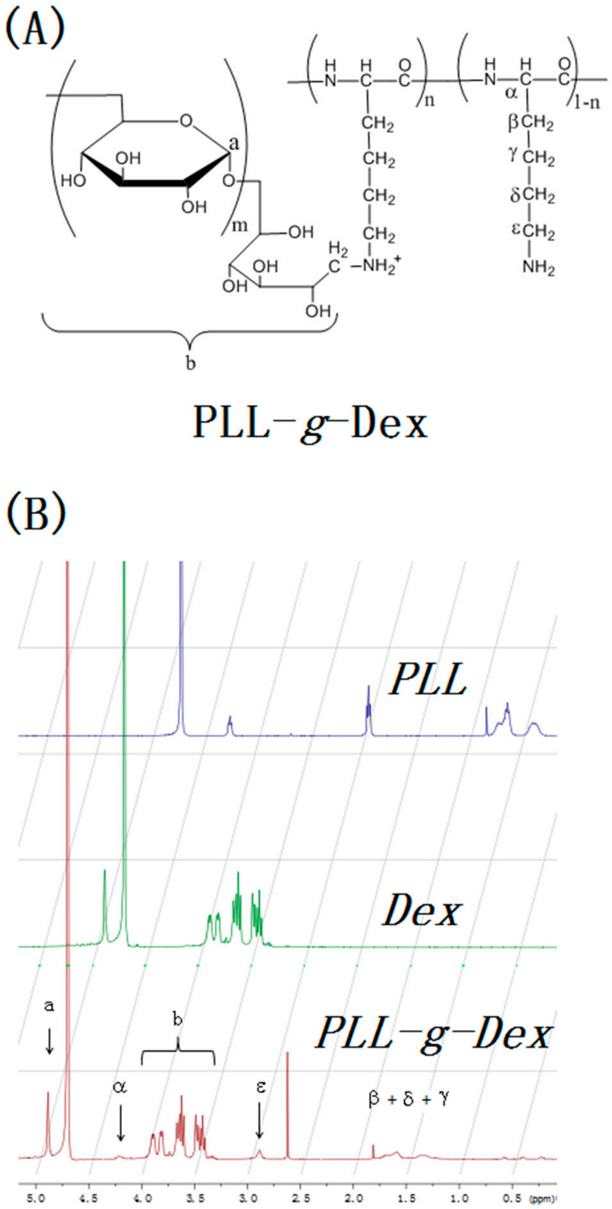


**Figure S1.** (**A**) Structural formula of poly(L-lysine)-graft-dextran (PLL-*g*-Dex) copolymer. (**B**) $^1$H-NMR spectra of PLL, Dex and PLL-*g*-Dex in $D_2O$. The dextran content of the copolymer was calculated from $^1$H-NMR signals assigned to PLL(ε -$CH_2$) and dextran($C_1$-H, a).

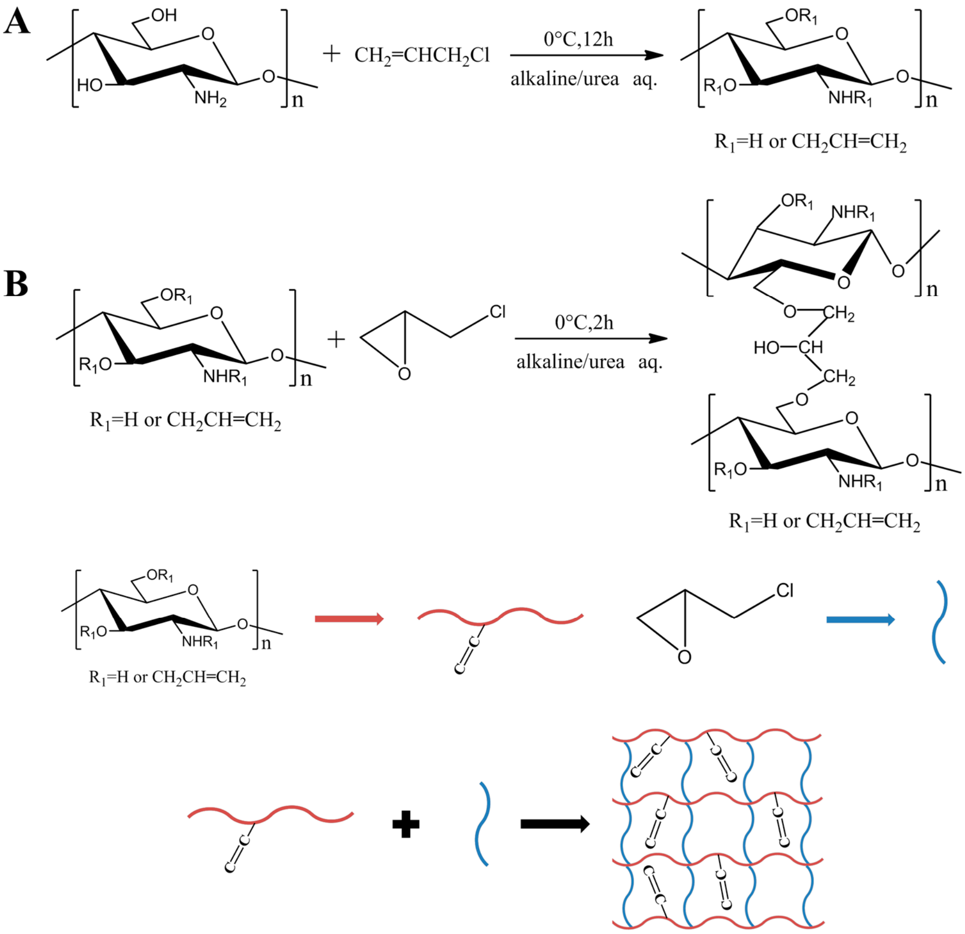


**Figure S2.** (**A**) Allylation of chitosan in KOH/LiOH/urea solution. (**B**) Schematic representation of ECH cross-link AC to prepare hydrogel film.

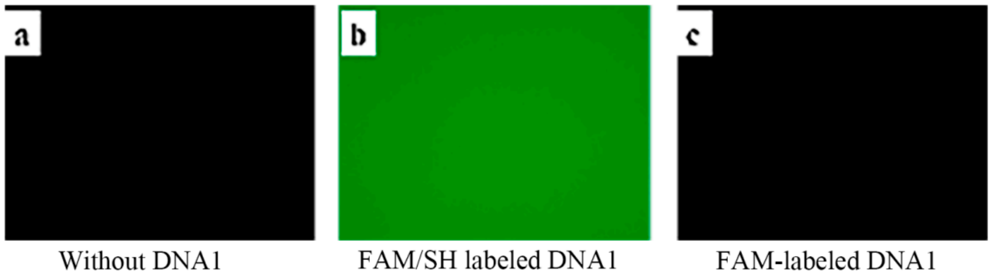


**Figure S3.** Fluorescent images the gels formed (**a**) without DNA1, (**b**) with FAM/SH-labeled DNA1, (**c**) with FAM-labeled DNA.
